# Sex trafficking awareness and associated factors among youth females in Bahir Dar town, North-West Ethiopia: a community based study

**DOI:** 10.1186/1472-6874-14-85

**Published:** 2014-07-16

**Authors:** Muluken Azage, Gedefaw Abeje, Alemtsehay Mekonnen

**Affiliations:** 1 Public Health Department, College of Medicine and Health Sciences, Bahir Dar University, Bahir Dar, Ethiopia

**Keywords:** Awareness, Sex trafficking, Youth females

## Abstract

**Background:**

Sex trafficking is a contemporary issue in both developed and developing countries. The number of trafficked women and young girls has increased globally. Females aged 18–25 are the most targeted group of trafficking. Although the problem is evident in Ethiopia, there are no studies that explored sex trafficking awareness among females. Therefore, the aim of this study was to assess sex trafficking awareness and associated factors among youth females in Bahir Dar town, North-West Ethiopia.

**Methods:**

A community based cross-sectional study design was employed to collect data from February 1^st^-30^th^ 2012 from a total of 417 youth females. The participants in the study were selected using systematic random sampling techniques. A structured Amharic questionnaire was used to collect data. Data were entered, cleaned and analyzed using SPSS 16.0. Descriptive statistics were used to describe data. Logistic regression analysis was used to identify factors associated with sex trafficking awareness.

**Result:**

Two hundred forty-nine (60%) of the participants reported that they had heard or read about sex trafficking. Television (64%), friends (46%) and radio (39%) were the most frequently mentioned sources of information about sex trafficking. About 87% and 74% of the participants mentioned friends and brokers respectively as mediators of sex trafficking. Having TV at home (AOR = 2. 19, 95% CI: 1.31-3.67), completing grade 10 or more (AOR = 2. 22, 95% CI: 1.18-4.17), taking training on gender issues (AOR = 3. 59, 95% CI: 2.11-6.10) and living together with parents (AOR = 3. 65, 95% CI: 1.68-7.93) were factors found associated with sex trafficking awareness.

**Conclusion:**

In this study, sex trafficking awareness was low among youth females. Having TV at home, living together with someone and being trained on gender issues were predictors of sex trafficking awareness. Therefore, providing education about sex trafficking will help to increase sex trafficking awareness among youth females.

## Background

The United Nation defines sex trafficking as “the recruitment, transportation, transfer, harboring, or receipt of persons, by means of the threat or use of force or other forms of coercion, of abduction, of fraud, of deception, of the abuse of power or of a position of vulnerability” for the purposes of sexual exploitation and for economic and other personal gains [1]. It is a contemporary public health issue of both developed and developing countries that violates human rights and has been described as a modern form of slavery [1,2].

According to the International Organization for Migration (IOM) and United Nation Office on Drugs and Crime report, the numbers of trafficked women have increased both in developed and developing countries [2,3]. In another study, girls aged 18–25 are mainly targeted by traffickers usually in poor socioeconomic areas [4,5]. It is difficult to estimate accurately the global prevalence of human trafficking due to its hidden nature [2]. But a recent estimate indicated that trafficking reaches between one and two million people each year worldwide; 60-70% of which are young girls [2,6]. The International Labor Organization (ILO) estimated that there are 4.5 million victims of forced sexual exploitation worldwide; 98% of whom are estimated to be women and girls [7].

Young girls are misguided by a well-organized group with promises of employment in some Western countries, North America, and Australia [2]. Sometimes, the girls themselves are aware and well informed that they would be engaged in prostitution abroad and pay huge sums of money to a mediator who assists them in obtaining passports or in transferring them illegally to the migrating country. The full cost associated with the migration would be covered by the mediator, with the hope that the woman would re-pay the loan from the proceeds of the prostitution abroad. Such agreement leads women to carry out various traditional rituals to ensure that they remain bonded to the mediator until they have been fully exploited financially [8].

A study conducted in Nigeria in 2004 showed that about 86.1% of students had heard about sex trafficking [9]. In another study conducted in Nigeria, 97.4% of the women reported that they had heard of women being taken abroad for commercial sex work. In this study, 47% of the young women believed that sex trafficking brings wealth and prosperity to the family [10]. About 76.5% of participants in above study believed that victims of sex trafficking were more likely to become infected with Sexually Transmitted Diseases (STDs). Another 56.7% believed that they would experience physical abuse and 56.6% thought they would have unwanted pregnancies [9].

Several studies reported that sex trafficking has impact on the physical, mental, social and psychological well being of women and girls [11-17]. Sex-trafficked women and girls are more likely to contract HIV and other STDs [18-21]. Studies done in India found that sex trafficking was a mode of entry into sex work [22,23]. These studies added that history of sex trafficking was associated with a greater vulnerability to violence and HIV risk behaviors [22,23]. About 88.3% of the women mentioned that sex trafficking had negative consequences to the life of women as it exposes them to STDs and HIV/AIDS [10].

Being out-of-school, unemployed, uneducated and unemployed parents, environmental and socio-cultural factors were factors mentioned as risk factors associated with sex trafficking in different studies [11,17,24,25]. A study done in Nigeria 77.2%, 68.4%, 56.1%, 44.5% of the participants reported poverty, unemployment, illiteracy and low social status respectively as factors associated with sex trafficking [11].

Likewise, thousands of teenage girls are shipped out of Ethiopia each year [5]. Recent reports showed that women and girls are also exploited in the sex trade after migrating for labor purposes [26]. Despite efforts made by the government and non-governmental organizations, victims of sex trafficking are increasing. Still, many women and young girls want to go abroad without knowing the situations there [26]. However, youth females’ awareness about sex trafficking is not yet well explored in Ethiopia, particularly in the study area. Credible evidence on awareness of sex trafficking at grass root level is important to formulate evidence based approaches for targeting preventative interventions against sexual trafficking. Therefore, this study was designed to assess sex trafficking awareness and associated factors among young females in Bahir Dar town, North-West Ethiopia.

## Methods

### Study design, area and population

A community based cross-sectional study design was employed from February 1^st^-30^th^, 2012. The study was conducted in Bahir Dar town, the capital city of Amhara National Regional State, which is located 565 kms North-West of Addis Ababa, Ethiopia. According to Central Statistics Agency (CSA) report of 2007 population census [27], the total population of the town was estimated 180,174 (87,160 males and 93,014 females). The majority of the population (96%) is Amhara by ethnicity and Orthodox Christian (90%) by religion [27]. All youth females aged 15–24 years who were living in the study area were the study population. Those youth females who lived at least six months in the town were included in the study.

### Sample size and sampling techniques

Single population proportional formula (n = [(Z_*α*/2_)^2^ P (1 - P)]/d^2^) was used to determine sample size based on the following assumptions: 95% level of confidence (Z_α/2_ = 1.96), the proportion of respondents who heard about sex trafficking in a previous study (p = 86%) (9), and margin of error (d = 4%). By considering 5% contingency, and 1.5 design effect, the final sample size was 456 youth females. Four urban kebeles (the lowest administrative level) of the total nine kebeles in the town were selected using a simple random sampling technique [28]. Proportional to size allocation was used in order to determine the required numbers of youth females from each kebele. Household was used as a sampling unit in this study. Households were selected using systematic random sampling. If there were more than one youth female in a household, lottery method was used to select one participant. If the selected youth was not available at home at the time of visit, revisit for the second time was made to contact the selected youth for interview.

### Data collection tool

A structured pretested Amharic questionnaire was used to elicit information about sex trafficking from the study participants (see Additional file 1). The questionnaire had two sections. The first section of the questionnaire was about socio-demographic characteristics of the youth females and their parents and the second section was on sex trafficking awareness. A youth female was classified as having awareness about sex trafficking if she reported that she had heard or read about sex trafficking that a woman who had been taken to another place or foreign countries for the purposes of sexual exploitation to gain money or other personal gains. As an indirect measure of the prevalence of sex trafficking in the area, a participant was asked whether or not she had been approached by someone that assists to go abroad.

Ten grade 10 complete females and two others were recruited as data collectors and supervisors for this study. One day training was given to data collectors and supervisors with particular emphasis on the objective of the study and methods of the survey.

### Data quality

The questionnaire was pretested to evaluate the face-validity and to ensure whether the study participants understood what the investigators intended to know and some modification of questions were made. Training was given to data collectors and supervisors on how to select household and study participants. Daily supervision was done by the principal investigator to check the completeness of the questionnaire. Female data collectors were used to get accurate information since most questions are gender sensitive.

### Data analysis

Data were entered, cleaned and analyzed with SPSS 16 software. Descriptive statistics such as mean and percentage were used to describe the data. Bivariate and multivariable logistic regression analyses were used to identify the predictors of sex trafficking awareness. Odds ratio with 95% confidence interval (CI) was calculated to identify predictors of sex trafficking awareness.

### Ethical clearance

Ethical clearance was taken from the ethical committee of the Bahir Dar University, College of Medicine and Health Sciences before data collection. Informed written consent was taken from the respective kebeles. Informed verbal consent and assent was obtained from study participants or parents after explaining the purposes of the study. Those who gave their consent and assent were interviewed. Confidentiality was insured by collecting the data anonymously.

## Results

A total of 417 youth females participated in this study. The response rate was 92%. About 64.7% of the study participants were in the age range of 20–24 years and 57.3% were single. The majority of the study participants (83.0%) were Orthodox Christian and 53% of the study participants had completed 10th grade and above. About 72.9% and 70.5% of the study participants had radio and television in their house respectively. The majority of study participants (33.6%) were students. One hundred twenty two participants took training on gender issues (Table 1).

**Table 1:** Socio-demographic characteristics of youth females in Bahir Dar Town, North-West Ethiopia, February 2012

**Variables**		**Frequency (n = 417)**	**Percent**
**Age**	15-19	147	35.3
	20-24	270	64.7
**Marital status**	Single	239	57.3
	Married	150	36.0
	Widowed	6	1.4
	Divorced	22	5.3
**Religion**	Orthodox	346	83.0
	Muslim	59	14.1
	Protestant	10	2.4
	Catholic	2	0.5
**Education level**	Illiterate	40	9.6
	Read and write	26	6.2
	Elementary	130	31.2
	Junior/secondary	142	34.1
	Above secondary	79	18.9
**Presence of radio at home**	Yes	304	72.9
	No	113	27.1
**Presence of TV at home**	Yes	294	70.5
	No	123	29.5
**With whom are you living**	With my parents	91	21.8
	Boyfriend/fiancé/husband	145	34.8
	Either mother or father only	68	16.3
	Relatives	61	14.6
	Alone	52	12.5
**Current job of the respondents**	No Job	117	28.1
	Student	140	33.6
	Some Job	76	18.2
	Not want to specify	84	20.1
**Had taken training on gender issues**			
	Yes	122	29.3
	No	295	70.7

Of all youth females, about 59.7% reported that they had read or heard about sex trafficking. About 64%, 46%, 37% and 17% of the study participants mentioned television, friends, radio and print materials respectively as sources of information. Friends and brokers were mentioned as mediators for sex trafficking by 87% and 74% of the study participants respectively. About 72%, 50%, 45%, and 18% of the study participants mentioned hoping for a better life elsewhere, unemployment, poverty and illiteracy respectively as reasons for being trafficked. About 71.4% of the participants reported that youth females ages greater than 25 years are vulnerable for sex trafficking. About 25% of the participants reported that they had been approached by someone else to assist them go abroad (Table 2).

**Table 2:** Sex trafficking awareness among youth females in Bahir Dar town, North-West Ethiopia, February 2012

**Variables**		**Responses**	
		**Frequency**	**Percent**
**Heard or read about sex trafficking**	Yes	249	59.7
	No	168	40.3
Source of information about sex trafficking* (n = 249)			
	Friends	114	45.9
	Radio	96	38.6
	Television	159	63.9
	News	42	16.9
	letter	9	3.6
	Others^a^		
Mediators for sex trafficking* (n = 249)			
	Friends	216	86.7
	Brokers	185	74.3
	Internet	22	8.8
	Others^b^	10	4.0
Reasons to be trafficked* (n = 249)			
	Poverty	111	44.6
	Unemployment	124	49.8
	Hope for better life elsewhere	180	72.3
	Illiteracy	45	18.1
	Low social status	9	3.6
	Entrapment	9	3.6
	False marriage	18	7.2
	Others^c^	7	2.8
**Which age groups of females are more vulnerable to be trafficked**			
	< 25 age group	298	71.4
	≥ 25 age group	17	4.1
	All age group	52	24.5
**Has someone approached you to assist you in going abroad?**			
	Yes	105	25.1
	No	212	74.9

*multiple responses are there, ^a^mini media, family, internet; ^b^relatives, strangers, tourists; ^c^family, divorce, family loss.

### Factors associated with awareness of sex trafficking

In bivariate logistic regression analysis, having radio and television at home, living with parents, boyfriend or fiancé or husband, and getting training on gender issues were significantly associated with awareness of sex trafficking. However, during multivariable logistic regression analysis, only having television at home; living with parents and boyfriend or fiancé or husband and getting training on gender issues showed statistically significant association with sex trafficking awareness. Completing grade 10 or above, living with either mother or father and alone did not show statistically significant association with sex trafficking awareness during multivariable logistic regression analysis (Table 3).

**Table 3:** Factors associated with awareness of sex trafficking among youth females in Bahir Dar Town, Northwest Ethiopia, February 2012

**Variables**		**Awareness of sex trafficking**			
				COR*(95% CI)	AOR*(95% CI)
		**Yes**	**No**		
**Age**	15-19	82	65	1.00	
	20-25	167	103	1.29 (0.86, 1.93)	
**Marital status**	Single	136	103	1.00	
	Married	94	56	1.27 (0.84,1.93)	
	Widowed	4	2	1.52 (0.27,8.43)	
	Divorced	15	7	1.62 (0.64,4.13)	
**Religion**	Orthodox	215	131	1.00	
	Muslim	31	28	0.68 (0.39, 1.16)	
	Protestant	6	4	0.26 (0.07,1.03)	
	Catholic	0	2	-	
**Education level**	Illiterate	22	18	1.00	1.00
	Read and write	19	7	2.22 (0.76,6.46)	1.41 (0.61,3.30)
	Elementary	81	49	1.35 (0.66,2.77)	0.81 (0.29, 2.30)
	Junior/secondary	75	65	0.92 (0.45,1.85)	1.27 (0.66,2.43)
	Above secondary	52	27	1.58 (0.72,3.43)	2.22 (1.18, 4.17)
**Presence of radio at home**	Yes	196	108	2.06 (1.33, 3.18)	
	No	53	60	1.00	
**Presence of TV at home**	Yes	191	103	2.08 (1.36,3.19)	2.19 (1.31, 3.67)
	No	58	65	1.00	1.00
**With whom are you living**	With my parents	66	25	4.22 (2.05, 8.71)	3.65 (1.68,7.93)
	Boyfriend/fiancé/husband	92	53	2.78 (1.45, 5.34)	3.46 (1.69,7.06)
	Either mother or father only	37	31	1.91 (0.92, 3.98)	2.31 (1.03,5.16)
	Relatives	34	27	2.02 (0.95,4.28)	2.86 (1.24,6.58)
	Alone	20	32	1.00	1.00
**Current job of the respondents**	No Job	65	52	1.00	
	Student	89	51	1.40 (0.85,2.31)	
	Some Job	51	35	1.63 (0.89,2.98)	
	Not want to specify	44	40	0.88 (0.50,1.54)	
**Taking training on gender issues**	Yes	95	27	3.22 (1.98,5.23)	3.59 (2.11, 6.10)
	No	154	141	1.00	1.00

*COR: crude Odds Ratio, AOR: Adjusted Odds Ratio.

Those whose educational status was grade 10 or above were 2.22 (AOR = 2. 22, 95% CI: 1.18-4.17) times more likely to be aware about sex trafficking than illiterates. Youth who had television at home were about 2.19 (AOR = 2. 19, 95% CI: 1.31-3.67) times more likely to be aware about sex trafficking compared to their counterparts. Youth who were living with parents, boyfriend or fiancé or husband, either mother and father and relatives were 3.65 (AOR = 3. 65, 95% CI: 1.68-7.93), 3.46 (AOR = 3. 46, 95% CI: 1.69-7.06), 2.31 (AOR = 2. 31, 95% CI:1.03-5.16) and 2.86 (AOR = 2. 86, 95% CI: 1.24-6.58) times more likely to be aware about sex trafficking respectively than those who were living alone. Youth trained on gender issues were 3.59 (AOR = 3. 59, 95% CI: 2.11-6.10) times more likely to be aware about sex trafficking compared to those youth females who did not take the training (Table 3).

## Discussion

Sex trafficking is one of the major contemporary public health issues in both developed and developing countries. Youth girls and children from low socioeconomic countries are more vulnerable for sex trafficking [2,11,24,25]. Still, many girls in many developing countries are waiting to go abroad without adequate information about the circumstance or situation there.

In this study, 60% of the study participants reported that they had heard or read about sex trafficking, which is lower than the studies done in Benin city, Nigeria in which about 97.4% of the respondents reported that they had heard or read about sex trafficking [10]. It is also lower than a study done among students in urban and rural schools of Delta and Edo states in Nigeria (86.1%) [9]. In this study, about 25% of young women had been approached by someone to assist them to go abroad, which is lower than the study done in Benin City, Nigeria (31.9%). This discrepancy may be due to the extent of the problem and sex trafficking promotion activities, and the cultural variation to discuss sensitive issues in the study areas. For instance, in Nigeria, there were well-organized programs that recruit young women and girls for sex trafficking at the time of the study [10]. A large proportion of young women in Benin City, Nigeria had been approached to assist them to travel abroad for sex trafficking [9,10]. Similarly, governmental and non-governmental organizations were involved in sex trafficking awareness promotion at the time of the study using print media and victims of sex trafficking were involved in experience sharing on television in Nigeria [10].

The sources of information about sex trafficking in this study were television, friends, radio and print materials which are consistent with the study done in Delta and Edo states of Nigeria [9]. In this study, about 29% of study participants had taken some training on gender issues which is more likely increase the awareness of sex trafficking.

Poverty was mentioned as a reason for sex trafficking in different regions of the world. Victims of sex trafficking are trafficked from relatively poorer areas to more affluent areas [2]. Poverty may lead women to leave their place for searching a job in other places [11,24-26]. In this study, about 45%, 50% and 72% of the study participants mentioned poverty, unemployment and hoping for a better life elsewhere respectively as reasons for sex trafficking. The above reasons are related to searching for economic opportunity which is consistent with other findings that support the role of poverty as underling cause of sex trafficking [17,21]. Similarly, other studies in South Asia, Nigeria and South Africa found that poverty was the underlying cause of sex trafficking [10,29-31].

In this study, friends and brokers were mentioned by 87% and 74% of the respondents respectively as mediators for sex trafficking. The above findings are consistent with the studies done in Ethiopia, Nigeria and South Africa [9,10,31,32].

In this study, about 71% of the respondents reported that young females aged less than 25 were more vulnerable than those aged greater than 25. This finding is consistent with a study done in South Asia (72%) [21].

In this study, completing grade 10 or above, having television at home, living with parents and boyfriend or fiancé or husband, living with either mother or father and relatives and taking training on gender issues were factors found associated with sex trafficking awareness. All the above factors were related to the accessibility of information on gender issues, including sex trafficking using different ways. Moreover, for the past decade international organizations, government and non-governmental organizations have been working on the empowerment of women to understand their rights and responsibilities in the communities through different media, workshops and training which leads females to have better awareness on reproductive issues and sexual rights including sex trafficking [33]. This study has also some limitations. Social desirability bias could be considered one potential challenge since some of the information was gender sensitive. Furthermore, the result may not be generalized beyond the study population itself since the study involved a small sample of a single, non-randomly selected city in Bahir Dar.

## Conclusion

The level of awareness of female youths about sex trafficking in this study was low. Having a television at home, completing grade 10 or above, living together with someone and taking training on gender issues were the predictors of sex trafficking awareness. Awareness creation on sex trafficking of youth females should be given through different approaches to increase the accessibility of information on sex trafficking. Moreover, further research is recommended to determine the magnitude and nature of sex trafficking in detail in the study area.

## Abbreviations

AOR: Adjusted odds ratio; CI: Confidence interval; IOM: International organization for migration; ILO: International labor organization; SPSS: Statistical package for social sciences; STDs: Sexually transmitted diseases.

## Competing interests

The authors declare that they have no competing interests.

## Authors’ contribution

MA designed the study, developed the questionnaire, supervised the data collection, analyzed the data and wrote the paper. GA supervised the data collection, contributed to the interpretation of the findings as well as the drafting and writing of the manuscript. AM trained data collectors, supervised the data collection, contributed to the interpretation of the findings and helped to draft the manuscript. All authors read and approved the final manuscript.

## Authors’ information

MA was graduated with BSc in Environmental Health and Master of Public Health in Environmental Health and working as lecturer at Department of Public Health, College of Medicine and Health Sciences, Bahir Dar University.

GA was graduated with BSc in Nursing, Master of Public Health in Reproductive health and working as lecturer at Department of Public Health, College of Medicine and Health Sciences, Bahir Dar University.

AM was graduated with BSc in Midwifery and Master of Public Health in Reproductive Health and working as lecturer at Department of Public Health, College of Medicine and Health Sciences, Bahir Dar University.

## Pre-publication history

The pre-publication history for this paper can be accessed here:

http://www.biomedcentral.com/1472-6874/14/85/prepub

## Supplementary Material

Additional file 1 Questionnaire to asses awareness of sex trafficking. Click here for file
