# Supplementary material for: Sex trafficking awareness and associated factors among youth females in Bahir Dar town, North-West Ethiopia: a community based study
Source: BMC Womens Health. 2014 Jul 16;14:85. doi: 10.1186/1472-6874-14-85 (PMC4118269; doi:10.1186/1472-6874-14-85)
Supplement: Additional file 1 — Questionnaire to asses awareness of sex trafficking. [file 1472-6874-14-85-S1.doc]

**Bahir Dar University**

**College of Medicine and Health Sciences**

**Questionnaire to asses awareness of sex trafficking**

**Introduction**

Good afternoon/Morning. I am ……………………………………………………….. . I am working in Bahir Dar University. The reason why I came to your home today is to collect data about young female’s awareness about sex trafficking. The study is conducted by Mr. Muluken Azage, Mr Gedefaw Abeje and S/r Alemtsehay Mekonnen who are academic staffs of Bahir Dar University, College of Medicine and Health Sciences.

You are selected to participate in this study. The selection was random. There are no risks if you are voluntary to participate in this study. There are no also incentives for your involvement in this study. But, the information, you provide, will help to know how young women are aware about sex trafficking. Sex trafficking is a growing public health problem which is affecting the lives of many women both nationally and globally. Your participation in this study is fully voluntary. Even, you can interrupt after we started the interview if you are not comfortable. You can also escape some questions for which you are not comfortable.

All the information you will give to me will be kept confidential. Your name and other identifiers will not be written on the questionnaire. All the information will be only used for research.

If you are voluntary to give information, I will ask you some socio-demographic and other questions. To complete the interview, I need about 30 minutes.

Do you agree to participate in this study?

1. Yes _______________(signature)
2. NO

Name of Kebele --------------------------------------

Name and Signature of data collector _______________________

Name and Signature of supervisor _______________________

Date and time interview started --------------------------------------

Date and time interview completed --------------------------------------

Part I. Socio-demographic characteristics of the respondents

| **S. no** | **Statement** | **Response** |
| --- | --- | --- |
| **101** | Age in years | ----- year |
| **102** | Marital status | 1. Single 2. Married 3. Widowed 4. Divorced |
| **103** | Religion | 1. Orthodox Christian 2. Muslim 3. Protestant 4. Catholic 5. Others (specify) …………. |
| **104** | Education status | 1. illiterate 2. Read and write 3. Elementary 4. junior/Secondary 5. Above secondary |
| **105** | Do you have a radio in your house? | 1. Yes 2. No |
| **106** | Do you have a TV in your house? | 1. Yes 2. No |
| **107** | With whom are you living now? | 1. Both parents 2. Boyfriend/fiancé/husband 3. Mother 4. Father 5. Alone 6. In –laws/step parents 7. Relatives 8. Others specify________ |
| **108** | What are you doing at present? | 1. Doing nothing 2. Schooling 3. Trading 4. Hair dressing 5. Teaching 6. Farming 7. Others specify ________ |
| **109** | Have you ever taken training on gender issues? | 1. Yes 2. No |

**Part II. Questions to assess awareness of sex trafficking among youth females**

| **S. no** | **Statement** | **Response** |
| --- | --- | --- |
| 201 | Have you heard or read about sex trafficking? | 1. Yes 2. No |
| 202 | If your answer for question 201 is yes, what is /are your source of information? Multiple response is possible | 1. Friend /relative 2. Radio 3. Television 4. News letter 5. Others(specify)…………………… |
| 203 | Do you know the ways/methods to undergoing sex trafficking? | 1. Yes 2. No |
| 204 | If your answer for question 203 is yes, What are the means for undergoing sex trafficking? More than one answer is possible | 1. Friends 2. Brokers 3. Internet 4. Other(specify) _______________ |
| 205 | Have you heard of women being taken abroad for prostitutions? | 1. Yes 2. No |
| 206 | If your answer to question number 205 is yes, what is your source of information? Multiple source is possible | 1. School 2. Media 3. Friend 4. News letter 5. Other _______ |
| 207 | What are the causes that facilitate sex trafficking? | 1. Poverty  2. Unemployment  3. Hope for better life elsewhere  4. Illiteracy  5. Low social status  6. Entrapment  7. False marriage  8. other(specify)_____________ |
| 208 | Do you know that there are people send women for prostitution to different countries to get money? | 1. Yes 2. No |
| 209 | Which age groups are most likely to be trafficked? | 1. People more than 25 years old? 2. People under 25 years old? 3. People of any age? 4. Don’t know/not sure |
| 210 | Has anybody approached you to assist you in going in other area or abroad? | 1. Yes 2. No |
| 211 | If yes for Q210, what kinds of jobs were you told you would do abroad? | 1. Hairdressing/nursing/cleaner 2. Was not told 3. Education 4. Prostitution 5. Trade/business 6. To marry 7. Learn skills 8. Visit 9. Meet parents/lived there |

**Thank you for your cooperation!!!**
